# Effects of high-level dietary distillers dried grains with solubles supplemented with multienzymes on growth performance, nutrient utilization, intestinal morphology, and pellet quality in broiler chickens

**DOI:** 10.14202/vetworld.2024.1943-1954

**Published:** 2024-08-31

**Authors:** Dingxing Jin, Elly Tugiyanti, Efka Aris Rimbawanto, Rosidi Rosidi, Titin Widiyastuti, Agus Susanto, Ismoyowati Ismoyowati

**Affiliations:** 1 Department of Animal Production, Faculty of Animal Science, Jenderal Soedirman University, Purwokerto, Indonesia; 2 New Hope Liuhe Indonesia Co., Ltd., Jakarta, Indonesia

**Keywords:** broiler chickens, distillers dried grains with solubles, growth performance, intestinal morphology, multienzyme, nutrient utilization, pellet quality

## Abstract

**Background and Aim:**

With the increasing cost of bulk raw materials and advancements in the feed enzyme industry, corn distillers dried grains with solubles (DDGS) have shown more opportunities for use in broiler diets. Supplementation with multiple enzymes could mitigate anti-nutritional factors in DDGS, enhance nutrient digestibility, and thereby increase its utilization in broiler diets, leading to reduced feed costs. This study evaluated the effects of multienzyme supplementation on growth performance, nutrient utilization, intestinal morphology, and pellet quality in broiler chickens fed diets containing conventional levels of DDGS (C-DDGS) and higher levels of DDGS (H-DDGS).

**Materials and Methods:**

A total of 800 1-day-old Cobb 500 chicks was assigned to four dietary treatments with eight replicates of 25 birds each: C-DDGS (5% DDGS in Starter and 10% in Grower), C-DDGS + Enzyme (C-DDGS diet supplemented with multienzyme), H-DDGS (10% and 20%) + Enzyme (H-DDGS diet supplemented with multienzyme, 10% DDGS in Starter and 20% in Grower), and H-DDGS (15% and 30%) + Enzyme.

**Results:**

The C-DDGS + enzyme diet increased (p < 0.05) body weight gain (BWG), reduced the feed conversion ratio, enhanced (p < 0.05) digestibility of dry matter (DM), crude protein, and hemicellulose (HC), and improved (p < 0.05) intestinal villus height and villus: crypt ratio of broilers. The H-DDGS (10% and 20%) + enzyme diet exhibited no difference in (p > 0.05) growth performance, nutrient digestibility (except HC), and intestinal morphological parameters, whereas the H-DDGS (15% and 30%) + enzyme diet decreased (p < 0.05) feed intake and BWG and reduced (p < 0.05) energy and DM digestibility by impact (p < 0.05) intestinal morphology compared with the C-DDGS enzyme-free diet. The H-DDGS diet had lower (p < 0.05) pellet hardness and poorer durability than the C-DDGS diet.

**Conclusion:**

Supplementing multienzyme in the C-DDGS (5% and 10%) diet improved growth performance from day 0 to 28 and diminished growth performance in the H-DDGS (15% and 30%) diet by influencing intestinal morphology and feed pellet quality in broiler chickens. In addition, when supplemented with multienzyme, the dietary DDGS level can be safely included at levels of 10% in 0–7 days and 20% in 8–28 days of age.

## Introduction

Soybean meal is the primary protein source in poultry diets, constituting over 90% of the developed protein ingredients. However, domestically cultivated soybeans in Indonesia only meet approximately 15% of the national demand, necessitating significant reliance on imported soybeans [1]. Recent developments, including export restrictions, escalated transportation costs, and depreciation of the Indonesian rupiah, have precipitated a notable escalation in domestic soybean meal prices, exerting a discernible impact on feed costs in Indonesia. Corn distillers dried grains with solubles (DDGS) have been recognized as a valuable substitute for corn and soybean meal in poultry feed because of their nutritional and economic benefits over the past few decades [2, 3]. Furthermore, the contemporary emphasis on renewable energy sources doubling the production of ethanol, generating substantial by-products like DDGS and augmenting its availability is particularly noteworthy in the US and other developing nations, including Indonesia.

DDGS is rich in crude protein (CP), fat, amino acids, B-group vitamins, biotin, and mineral compounds, and it has higher phosphorus bioavailability, with all nutrients except starch increasing approximately 3-fold compared with those in corn because starch is converted to ethanol and CO_2_ during the fermentation process [4, 5]. About 6%–10% is recommended for DDGS in starter broiler diets, while 12%–16% is suggested in grower/finisher diets [6–10]. However, the concentration of components in DDGS leads to a significant increase in anti-nutritional factors, especially the amount of non-starch polysaccharides (NSP), coupled with protein denaturation and lower amino acid digestibility caused by the heat-drying process, making DDGS impractical for high-level incorporation in broiler diets [4, 11]. DDGS comprises 25%–30% NSP and is predominantly composed of arabinoxylan and cellulose, in which arabinoxylan and other water-soluble hemicellulose (HC) contribute to the heightened viscosity of digesta in the gut, and water-insoluble cellulose cannot be digested by poultry due to the lack of endogenous cellulase. These factors collectively reduce overall diet digestion and affect the absorption of nutrients [12, 13].

The addition of an exogenous single enzyme or multi-enzyme complex mitigates the negative effects of DDGS and enhances economic returns. A single enzyme, particularly xylanase, has proven effective in enhancing nutrient digestibility and growth performance in broiler diets containing DDGS [14]. Moreover, a study indicated that the combined use of xylanase and phytase in broiler diets could overcome the limitations associated with conventional DDGS (C-DDGS) levels and can be included at a level of 12% in the starter and 18% in finisher periods without any detrimental effects [15], suggesting that high levels of DDGS (H-DDGS) can be used in broiler diets with appropriate enzyme supplementation. In addition, supplementing multienzyme complexes (NSPase or combined protease) in diets containing DDGS has been shown to contribute to the reduction of intestinal digesta viscosity [16], modulating intestinal microbiota [17], producing prebiotic xylo-oligosaccharides from xylans with xylanases [18], and ultimately improving digestibility and benefits in weight gain [7, 19]. Another obstacle affecting the high-level utilization of DDGS in broiler diets is the high variability resulting from corn varieties, industrial practices, drying processes, and the proportion of soluble additives, all of which interfere with the nutritional composition and bioavailability of nutrients [20, 21]. Although exogenous enzyme supplementation can mitigate some of the effects caused by anti-nutritional factors and the variability of DDGS, a single enzyme or several combinations of enzymes may not completely overcome the nutritional challenges associated with a diet containing H-DDGS. Comprehensive multienzyme supplementation in diets containing high DDGS levels requires further evaluation.

Based on previous research [6, 10], this study focused on diets with 5% and 10% DDGS and then raised the percentage to assess the potential for enhancing the value of a H-DDGS diet through the addition of an extensive multienzyme complex (xylanases, β-glucanases, mannanases, cellulose, protease, and phytase.). Our study investigated the ideal dosage of DDGS and its multienzyme supplementation effects on broiler growth performance, nutrient digestibility, intestinal morphology, pellet quality, and potential cost savings by replacing soybean meal with these treatments.

## Materials and Methods

### Ethical approval

This study was approved by the Animal Ethics Committee of Jenderal Soedirman University (No. 1923/UN.23.14/PN.01/2023).

### Study period and location

The study was conducted from July 2023 to September 2023. Fieldwork was conducted in the city of Kuningan, Department of Central Java, Cirebon County, located at an altitude of 768 m above sea level, with an average temperature of 25°C.

### Experimental diets

The experiment using a single-factor experimental design was conducted in two stages; starter 1–7 days, and finisher, 8–28 days, and was designed into four treatments: C-DDGS (5% DDGS in Starter and 10% in Grower), C-DDGS + Enzyme (C-DDGS diet supplemented with multienzyme), H-DDGS (10% and 20%) + Enzyme (H-DDGS diet supplemented with multienzyme, 10% DDGS in Starter and 20% in Grower) and H-DDGS (15% and 30%) + Enzyme (H-DDGS diet supplemented with multienzyme, 15% DDGS in Starter and 30% in Grower). The treatment diets were based on corn and soybean meal and were developed according to the nutritional requirements of Cobb [22] and followed the New Hope Indonesia Nutritional recommendation standard for broiler chickens. All experimental groups had similar raw material and nutrient contents. DDGS used in this experiment was imported from the US, and its main nutrients were as follows: Dry matter (DM), 90%; CP, 28.4%; crude fat, 7.5%; crude ash, 4.7%; crude fiber (CF), 7.8%; neutral detergent fiber (NDF), 38.2%; acid detergent fiber (ADF), 14.8%; and apparent metabolizable energy, 2490 Kcal/kg. The multienzyme complex used in this experiment was provided by VTR (VTR Biotech Co. Ltd, Guangzhou, China). The product included NSP enzymes (hemicellulase: xylanases 50,000 U/g, *β*-glucanases 50,000 U/g and mannanases 5,000 U/g, and cellulase 10,000 U/g), protease at 20,000 U/g, and phytase at 5000 U/g. In addition, Titanium dioxide (TiO) was added to all diets during both the starter and grower periods at an inclusion rate of 0.4% and used as an indigestible marker to determine nutrient digestibility. All diets were produced by New Hope Liuhe Indonesia Co., Ltd. (Cirebon, West Jawa, Indonesia) and pelleted using a pellet press machine (K15 model, FAMSUN, Jiangsu, China) equipped with a compression ratio (die effective length to diameter ratio) equal to 16 (48:3.2 mm). A crusher was used to crush the pellets into crumbles for the starter diet. The composition of the treatment diets is presented in Table-1.

**Table-1:** Dietary ingredients and nutrient composition of the experimental diets (as-fed basis).

Ingredients, kg/t	C-DDGS (5% and 10%)		C-DDGS (5% and 10%) + Enzyme		H-DDGS (10% and 20%) + Enzyme		H-DDGS (15% and 30%) + Enzyme	

	1–7 day	8–28 day	1–7 day	8–28 day	1–7 day	8–28 day	1–7 day	8–28 day
Corn	389.0	420.5	388.4	419.9	361.0	364.8	335.5	308.0
Wheat	150.0	150.0	150.0	150.0	150.0	150.0	150.0	150.0
Refined palm oil	27.0	29.0	27.0	29.0	31.0	37.0	34.0	46.0
Soybean meal, 46%	338.0	259.0	338.0	259.0	311.0	205.0	283.0	152.0
DDGS	50.0	100.0	50.0	100.0	100.0	200.0	150.0	300.0
Lines tone	11.0	8.0	11.0	8.0	11.0	9.0	12.0	10.0
Di calcium phosphate	20.0	18.0	20.0	18.0	20.0	17.0	19.0	16.0
Sodium bicarbonate	1.2	1.1	1.2	1.1	1.1	1.0	1.0	0.8
Salt	3.0	2.7	3.0	2.7	2.7	2.2	2.5	1.7
D, L-methionine, 99%	3.1	3.0	3.1	3.0	3.1	3.0	3.1	3.0
L-lysine HCL,98%	2.8	3.6	2.8	3.6	3.4	4.9	4.0	6.1
L-threonine, 99%	1.3	1.4	1.3	1.4	1.5	1.7	1.6	1.9
L-threonine, 99%	0.1	0.2	0.1	0.2	0.1	0.3	0.2	0.4
Choline chloride, 60%	1.0	1.0	1.0	1.0	1.0	1.0	1.0	1.0
Premi×^1^	2.5	2.5	2.5	2.5	2.5	2.5	2.5	2.5
Multienzyme^2^			0.6	0.6	0.6	0.6	0.6	0.6
Total	1000	1000	1000	1000	1000	1000	1000	1000
Calculated nutrients								
Dry matter (%)	88.79	88.60	88.79	88.60	88.81	88.64	88.82	88.73
Metabolizable energy/Kcal/kg	2920.9	2990.2	2920.9	2990.2	2922.9	2993.9	2921.7	2991.6
Crude protein content (%)	22.58	20.54	22.58	20.54	22.57	20.52	22.52	20.53
Calcium (%)	0.98	0.81	0.98	0.81	0.98	0.83	1.00	0.85
Total phosphorus (%)	0.76	0.72	0.76	0.72	0.77	0.73	0.77	0.79
Available phosphorus (%)	0.45	0.40	0.45	0.40	0.45	0.40	0.45	0.40
Fat (%)	4.86	5.33	4.86	5.33	5.42	6.46	5.89	7.66
Ash (%)	6.69	5.93	6.69	5.93	6.69	5.96	6.70	6.27
Crude fiber percentage	2.97	3.17	2.97	3.17	3.15	3.55	3.34	3.91
Na (%)	0.18	0.18	0.18	0.18	0.18	0.18	0.18	0.19
Cl (%)	0.30	0.30	0.30	0.30	0.30	0.30	0.30	0.31
Digestible lysine (%)	1.26	1.14	1.26	1.14	1.25	1.15	1.25	1.15
Digestible methionine (%)	0.61	0.58	0.61	0.58	0.61	0.58	0.61	0.58
DMet (%) + DCys (%)	0.93	0.88	0.93	0.88	0.93	0.88	0.93	0.87
Digestible threonine (%)	0.86	0.78	0.86	0.78	0.87	0.78	0.86	0.77
Digestible tryptophan (%)	0.22	0.20	0.22	0.20	0.22	0.20	0.22	0.19
Digestible isoleucine content (%)	0.86	0.86	0.86	0.86	0.84	0.84	0.81	0.81
Digestible valine (%)	0.97	1.14	0.97	1.14	0.95	1.03	0.94	0.92
Digestible leucine content (%)	1.68	0.75	1.68	0.75	1.69	0.71	1.70	0.66
Digestible arginine (%)	1.35	1.57	1.35	1.57	1.30	1.60	1.24	1.62

1 Premix supplied per kg of diet: Vitamin A,10000 IU; Vitamin D, 5 MIU; Vitamin E, 100 mg; Vitamin K, 3 mg; riboflavin 10 mg; nicotinic acid, 55 mg; calcium pantothenic acid, 20 mg; folic acid, 2 mg; thiamine 3 mg; riboflavin, 10 mg; biotin, 0.3 mg; pyridoxine, 5 mg; cobalamin, 25 mg; manganese, 100 mg, iron, 40 mg, zinc, 100 mg, copper 15 mg, iodine 1 mg, selenium 0.4 mg.

2 Multienzyme: The recommended level of supplementation of this complex enzyme was 600 g/ton, and the dietary enzyme activity for xylanase, β-glucanases, mannanase, cellulase and protease, and phytase was 5000, 5000, 500, 1000, and 5000 U/kg, respectively, and 500 FTU/kg in feed, respectively. DDGS=Corn distillers dried grains with solubles, C-DDGS=Conventional distillers dried grains with solubles level, H-DDGS=High distillers dried grains with solubles level

### Broiler management

A total of 800 1-day-old Cobb 500 chicks (half male and half female, average body weight 42 ± 1.02 g) was obtained from New Hope poultry hatchery (Cirebon) and randomly allocated to four treatments, each of which had eight pens with 25 chicks per pen. All chicks were raised in floor pens (2.5 m × 1.2 m, 3 m^2^) with fresh rice hulls (10 cm deep) and provided *ad* *libitum* access to diets and water with controlled ventilation. The temperature was maintained at 33 on day 1, gradually reduced until reaching 24°C–25°C by the 3^rd^ week and maintained thereafter. Lighting and feeding management procedures were implemented in accordance with the management standards of Cobb [22]. The chicks and feed were weighed on a pen basis weekly, enabling the determination of weekly feed intake (FI), body weight gain (BWG), and feed conversion ratio (FCR). FCR was calculated as the ratio of FI per pen to the average weekly weight gain per pen for the corresponding periods. Mortality was also recorded daily; the total mortality of the 7-day period and the entire experimental duration was calculated. All experiments were conducted at New Hope Indonesia’s Cirebon Broiler Experimental Farm (Cirebon), and all procedures followed the company’s animal experimental management regulations.

### Sample collection

On day 28, four broilers (half male and female) from each replication (32 birds per treatment) were randomly selected for slaughter. Two of them were used to collect ileal samples, and the other two were used to collect intestinal tissue samples. A section of ileum was removed by cutting it from Meckel’s diverticulum to the ileocecal junction to collect ileal digesta. A 50-mL syringe filled with room-temperature (25°Χ) deionized water was inserted into one end of the ileum to carefully flush out digesta from the gut into a 10-cm-diameter Petri dish. The digesta from each replicate were pooled and freeze-dried to calculate the coefficient of ileal apparent digestibility of the diet components. Intestinal samples (duodenum, jejunum, and ileum) were also collected from the other two birds to determine intestinal histomorphology. Briefly, 3-cm segment duodenum (from the midpoint of the duodenum), jejunum (from the midpoint between the bile duct entry and Meckel’s diverticulum), and ileum (from the midpoint of Meckel’s diverticulum and ileocecal junction) were collected and gently rinsed with a saline solution to remove the intestinal content. Tissue samples were preserved and fixed in 10% buffered formalin phosphate solution (100 mL 40% formaldehyde, 4 g phosphate, 6.5 g dibasic sodium phosphate, and 900 mL distilled water) for 18–24 h. The samples were prepared, sectioned, and stained using the method of Kim *et al*. [23] and were examined under a light microscope. Villi height and crypt depth (CD) were measured in triplicate using Image Raster 4.0.5 Software (PT. Miconos Transdata Nusantara, Yogyakarta, Indonesia). Villus height (VH, μm) was determined from the top of the villus to the crypts of Lieberkühn, and (CD, μm) was measured as the depth of invagination between adjacent villi. All morphological characteristics were analyzed in 10-μm increments.

Feed samples (pellets) from each treatment were collected from the discharge port of the granulator after pelleting to determine pellet hardness. Crumble samples were collected from the observation port of the cooler after the crushing process to determine powder content (For the starter feed, pellet samples were still used to measure the powder content in the Grower feed). In addition, to investigate pellet durability during transportation, feed samples from each replication were collected from the experimental farm (located 60 km from the feed mill) immediately to determine powder content. All feed samples from each replication, collected from different locations, were thoroughly mixed for subsequent nutritional analyses. Pellet hardness (measured in Newton) was measured in 100 pellets of each replication sample using a Brookfield CT3 10,000-g texture analyzer (Middleborough, USA) with a cylindrical probe number 3 and set for compression testing (2 mm target test and 1.5 mm/s speed). The powder content was determined using a 14-mesh screen. The method involved taking a 1.2 kg of sample, dividing it into two parts using the quartering method, and placing 600 g on a screen. The sample was sifted by hand for 1 min (110–120 vibrations/min), and the weight of the undersize fraction was divided by the initial sample weight and then multiplied by 100.

### Chemical analysis

The components in the samples were determined using standard procedures methods (method 930.15; [24]) for DM (DM; #934.01), CP (#976.05), crude fiber (#962.09), and ether extracts (EE) (#920.39). Gross energy was measured using an oxygen bomb calorimeter (Model 6300, PARR, Moline, IL). The NDF and ADF contents were measured as described by Van Soest *et al*. [25]. HC was calculated as the difference between NDF and ADF. The concentrations of TiO_2_ in samples were determined using the method reported by Fowler *et al*. [26]. The concentrations of sulfur in the feed of each treatment group and DDGS were determined by ion chromatography, as described by Novo *et al*. [27].

### Statistical analysis

Results from each treatment were analyzed using the general linear model procedure (GLM) of SAS 9.4 (SAS Institute, Cary, NC, USA) [28] with a completely randomized design. One-way analysis of variance was used to assess the main effects, and Tukey’s honestly significant difference test was used to assess significant differences among treatment means. Significance was considered at the level of p < 0.05 (0.05 < p < 0.10 was considered a trend toward significance). All test data are presented as means standard error of the mean.

## Results

### Growth performance

All growth performance data, including FI, BWG, FCR, and mortality, are presented in Table-2. Multienzyme supplementation in C-DDGS (5% and 10%) did not affect (p > 0.05) FI, BWG, and FCR in starter (day 1–7), FI in the grower (day 8–28), or the overall period (day 1–28). However, a significant improvement (p < 0.05) in BWG and FCR was observed in the grower and overall period when supplemented with multienzyme in the C-DDGS (5% and 10%) diet compared to no supplementation. At the same time, there were no significant differences (p > 0.05) in growth performance parameters between broiler chickens fed an H-DDGS (10% and 20%) diet supplemented with multienzyme and those fed a C-DDGS (5% and 10%) enzyme-free diet during any period. However, the H-DDGS (15% and 30%) diet supplemented with multienzyme significantly decreased (p < 0.05) BWG in all growth stages and FI during the growth period compared with the C-DDGS (5% and 10%) enzyme-free diet. In addition, despite supplementation with the same level of multienzyme, high dietary levels of DDGS (10% and 20% and 15% and 30%) significantly decreased (p < 0.05) BWG, and the 15% and 30% level of DDGS significantly decreased (p < 0.05) FI and impaired FCR at 8–28 days and 1–28 days compared with the 5% and 10% DDGS diet. This effect was not observed during the 1^st^ week. A tendency toward increased mortality (p = 0.068) was observed with increased dietary DDGS levels.

**Table-2:** Effects of high-level dietary DDGS supplemented with multiple enzymes on broiler growth performance.

Age (days)	Parameters	Treatments				SEM	p-value

		C-DDGS^1^ (5% and 10%)	C-DDGS (5% and 10%) + Enzyme	H-DDGS (10% and 20%) + Enzyme	H-DDGS (15% and 30%) + Enzyme		
1–7	FI (g/bird)	150.9	150.1	146.2	147.0	3.17	0.398
	BWG (g/bird)	161.6^a^	162.4^a^	159.6^ab^	159.2^b^	2.10	0.041
	FCR	0.934	0.924	0.916	0.942	0.158	0.405
8–28	FI (g/bird)	2226.6^a^	2219.6^a^	2195.2^ab^	2167.2^b^	14.84	0.004
	BWG (g/bird)	1478.8^b^	1503.8^a^	1468.2^b^	1433.6^c^	10.65	0.001
	FCR	1.506^a^	1.476^b^	1.496^ab^	1.510^a^	0.010	0.022
1–28	FI (g/bird)	2377.6^a^	2369.6^a^	2341.4^ab^	2314.2^b^	14.44	0.002
	BWG (g/bird)	1640.4^b^	1666.2^a^	1627.8^b^	1589.8^c^	9.75	<0.001
	FCR	1.449^ab^	1.422^c^	1.438^bc^	1.456^a^	0.008	0.006
	Mortality (%)	4.76	4.62	6.36	7.23	1.069	0.068

1 C-DDGS=Conventional distillers dried grains with solubles level, H-DDGS=High distillers dried grains with solubles level, FI=Feed intake, BWG=Body weight gain, FCR=Feed conversion ratio. ^a,b,c^Means within a column with different superscript letters differ significantly (p < 0.05), SEM=Standard error of the mean

### Nutrient digestibility

The C-DDGS (5% and 10%) diet supplemented with multienzyme significantly increased (p < 0.05) the digestibility of DM, CP, and HC by 4.65%, 4.83%, and 6.1%, respectively. However, it did not affect (p > 0.05) the apparent ileal digestible energy (AIDE) compared to the diet without multienzyme supplementation (Table-3). In contrast, H-DDGS (15% and 30%) supplemented with multienzyme significantly decreased (p < 0.05) AIDE levels by 111.8 Kcal/kg and digestibility of DM by 6.0% but increased (p < 0.05) the digestibility of HC by 10.56% and did not affect (p > 0.05) digestibility of CP and EE. At the same time, there were no significant differences (p > 0.05) in the AIDE levels and digestibility of DM, CP, and EE between broiler chickens fed an H-DDGS (10% and 20%) diet supplemented with multienzyme and those fed a C-DDGS (5% and 10%) enzyme-free diet. However, the digestibility of HC was significantly higher (p < 0.05) in the H-DDGS (10% and 20%) diet. H-DDGS supplemented with a multienzyme tended to increase the digestibility of CF (p = 0.091). The digestibility of EE was not significantly affected (p > 0.05) by DDGS levels or the supplemented multienzyme.

**Table-3:** Effect of high-level dietary DDGS supplemented with multiple enzymes on the nutrient digestibility of broiler.

Nutrients (%)	Treatments				SEM	p-value

	C-DDGS^1^ (5% and 10%)	C-DDGS (5% and 10%) + Enzyme	H-DDGS (10% and 20%) + Enzyme	H-DDGS (15% and 30%) + Enzyme		
AIDE (Kcal/kg)	3109.4^a^	3125.2^a^	3091.2^a^	2997.6^b^	28.36	0.002
DM (%)	72.66^b^	76.04^a^	70.98^b^	68.4c	1.41	<0.001
CP (%)	69.06^b^	72.40^a^	67.38^b^	66.76^b^	1.67	0.017
EE (%)	80.66	80.37	81.21	82.42	1.88	0.680
CF (%)	11.85	12.61	13.65	13.57	0.76	0.091
HC (%)	39.78^b^	42.41^a^	45.62^a^	44.60^a^	1.16	0.001

1 C-DDGS=Conventional level of distillers dried grains with solubles, H-DDGS=High level of distillers dried grains with solubles, AIDE=Apparent ileal digestible energy, CP=Crude protein, DM=Dry matter, EE=Ether extract, CF=Crude fiber, HC=Hemicellulose. ^a,b,c^Means within a column with different superscript letters differ significantly (p < 0.05), SEM=Standard error of the mean

### Intestinal morphology

As shown in Table-4, the VH and villus/crypt in the duodenum, jejunum, and ileum were significantly increased (p < 0.05) by multienzyme supplementation in the C-DDGS diet. However, it did not affect (p > 0.05) the CD in small intestine. The H-DDGS (15% and 30%) diet supplemented with multienzyme had a significantly negative effect (p < 0.05) on all intestinal morphological parameters in the duodenum and decreased (p < 0.05) VH and villus/crypt in the jejunum and ileum compared with the C-DDGS (5% and 10%) enzyme-free diet. The H-DDGS (10% and 20%) multienzyme-supplemented diet had no effect (p > 0.05) on intestinal morphological parameters in the entire small intestine compared with the C-DDGS enzyme-free diet. In the three multienzyme-supplemented treatments, intestinal VH, CD, and villus/crypt significantly decreased (p < 0.05) with increasing dietary DDGS levels.

**Table-4:** Effect of high-level dietary DDGS supplemented with multiple enzymes on the intestinal morphology of broiler.

Parameters	Treatments				SEM	p-value

	C-DDGS^1^ (5% and 10%)	C-DDGS (5% and 10%) + Enzyme	H-DDGS (10% and 20%) + Enzyme	H-DDGS (15% and 30%) + Enzyme		
Duodenum						
Villus height (μm)	1393.7^b^	1462.1^a^	1374.8^b^	1296.6^c^	31.67	<0.001
Crypt depth (μm)	172.8^bc^	153.2^c^	184.2^b^	192.6^a^	9.67	0.005
Villus: Crypt (μm)	8.15^b^	9.60^a^	7.52^ab^	6.73^c^	0.42	<0.001
Jejunum						
Villus height (μm)	870.7^b^	953.6^a^	851.8^b^	803.3^c^	20.21	<0.001
Crypt depth (μm)	145.9^ab^	128.2^b^	155.2^ab^	163.6^a^	11.08	0.032
Villus: Crypt (μm)	6.08^b^	7.55^a^	5.55^bc^	4.93^c^	0.41	<0.001
Ileum						
Villus height (μm)	649.7^b^	702.6^a^	622.8^bc^	582.3^c^	26.49	0.003
Crypt depth (μm)	126.3^ab^	110.5^b^	136.2^a^	144.8^a^	11.4	0.047
Villus: Crypt (μm)	5.29^b^	6.52^a^	4.63^bc^	4.06^c^	0.49	<0.001

1 C-DDGS=Conventional distillers dried grains with solubles level, H-DDGS=High distillers dried grains with solubles level. ^a,b,c^Means within a column with different superscript letters differ significantly (p < 0.05) SEM=Standard error of the mean

### Dietary pellet quality, sulfur content, and economic benefits

In the starter feed, a diet containing 5% and 10% DDGS with multienzyme did not affect (p > 0.05) pellet hardness, feed powder content in the feed mill, or farm and sulfur content compared with a 5% DDGS enzyme-free diet (Table-5). The 15% DDGS diet supplemented with enzyme significantly decreased (p < 0.05) pellet hardness by 13.66% and increased (p < 0.05) powder content on the farm by 46.39% and sulfur content. In grower feed, there were no significant differences (p > 0.05) in pellet hardness, powder content, and sulfur content between the 10% DDGS and 5% DDGS diets. The H-DDGS (20% and 30%) diet with multienzyme supplementation significantly reduced pellet hardness and increased (p < 0.05) feed powder content on the farm and sulfur content compared with the C-DDGS diet. The levels of DDGS in the diet or the supplementation of enzymes did not significantly affect (p > 0.05) feed powder content at the feed mill. The sulfur content in the 15% DDGS diet was significantly higher (p < 0.05) than that in the other diets in starter feed, and the 20% and 30% DDGS diets had higher (p < 0.05) sulfur content than the 10% DDGS diet in grower feed, with the 30% DDGS diet exhibited the highest (p < 0.05) sulfur content.

**Table-5:** Effect of high-energy dietary DDGS supplemented with multiple enzymes on dietary pellet quality and sulfate content.

Parameters	Treatments				SEM	p-value

	C-DDGS^1^ (5% and 10%)	C-DDGS (5% and 10%) + Enzyme	H-DDGS (10% and 20%) + Enzyme	H-DDGS (15% and 30%) + Enzyme		
Starter feed (Crumble)	DDGS 5%	DDGS 5%	DDGS 10%	DDGS 15%		
Pellet hardness (n)	37.34^a^	36.91^a^	35.66^a^	32.24^b^	1.56	0.019
Powder content 1 (%)^2^	5.68	5.22	6.76	7.31	1.08	0.238
Powder content 2 (%)^3^	13.58^b^	13.72^b^	16.21^b^	19.88^a^	1.57	0.003
Sulfur content (mg/kg)	2474.3^b^	2502.7^b^	2806.5^b^	3270.7^a^	189.9	0.002
Grower feed (Pellet)	DDGS 10%	DDGS 10%	DDGS 20%	DDGS 30%		
Pellet hardness (n)	35.22^a^	34.54^a^	32.24^b^	27.68^c^	1.95	0.002
Powder content 1 (%)	4.18	3.92	5.46	6.4	1.12	0.135
Powder content 2 (%)	8.58^c^	8.72^c^	12.01^b^	15.08^a^	1.42	<0.001
Sulfur content (mg/kg)	2952.7^c^	2991.8^c^	3795.5^b^	4470.7^a^	240.5	<0.001

1 C-DDGS=Conventional distillers dried grains with solubles level, H-DDGS=High level of distillers dried grains with solubles;

2 Powder content 1, the feed powder content detected at the feed mill;

3 Powder content 2: The feed powder content was detected at the experiment farm. ^a,b,c^Means within a column with different superscript letters differ significantly (p < 0.05) SEM=Standard error of the mean

The formula and unit meat production costs of the treatment diets are presented in Supplementary Table-1. The formulation cost of the C-DDGS diet was USD 0.4475/kg. This cost decreased by USD 0.0098/kg for H-DDGS (10% and 20%) and USD 0.0193/kg for H-DDGS (15% and 30%) diet. After incorporating the costs of multienzyme and feed manufacturing, the C-DDGS (5% and 10%) + enzyme combination had the highest overall treatment feed cost of 0.4709 USD/kg, whereas the H-DDGS (15% and 30%) + enzyme combination had the lowest overall feed cost of 0.4516 USD/kg. The final feed cost for producing 1 kg of meat was approximately calculated by multiplying the overall cost of the treatment diet by the feed consumption per kg of meat produced (FCR). Compared with the C-DDGS diet, the final meat production costs were lower in the other treatment groups, with the H-DDGS + enzyme group exhibited the lowest meat production cost.

**Supplementary Table-1:** Analysis of formula cost and unit meat production cost of treatment diets.

Parameters	Treatments			

	C-DDGS^1^ (5% and 10%)	C-DDGS (5% and 10%) + Enzyme	H-DDGS (10% and 20%) + Enzyme	H-DDGS (15% and 30%) + Enzyme
Formulation cost (USD/kg)^2^	0.4475	0.4475	0.4377	0.4282
Multienzyme preparation cost (USD/kg)		0.0080	0.0080	0.0080
Feed manufacturing cost (USD/kg)	0.0154	0.0154	0.0154	0.0154
Total feed cost (USD/kg)	0.4629	0.4709	0.4611	0.4516
Feed consumed/kg of meat gain (kg)	1.449	1.422	1.438	1.456
Total feed cost/kg of meat gain (USD/kg)^3^	0.6707	0.6696	0.6629	0.6574
Difference versus control (USD/kg)	-	−0.0011	−0.0077	−0.0041

1 C-DDGS=Conventional distillers dried grains with solubles level, H-DDGS=High level of distillers dried grains with solubles,

2 Main raw material prices (USD/kg): Corn, 0.3991; Wheat, 0.3316; Soybean meal, 0.5526; DGS, 0.3377; Multienzyme preparation, 13.3054.

3 Total Feed cost/kg meat gain=total feed cost × feed consumed/kg meat gain

## Discussion

### Effects of H-DDGS supplemented with multiple enzymes on broiler growth performance

A previous study [6] documented that diets containing C-DDGS are beneficial for the growth and reproductive performance of broiler chickens. Early results indicated that diets containing 6% DDGS in the starter period (days 1–16) and 12% DDGS in growers (days 17–31) did not affect weight gain and gain: feed, suggesting that this addition level could be considered conventional as an alternative source of energy and protein in poultry diets with other feed components [6]. Min *et al*. [29] reported an increase in average daily gain and a decrease in FCR for broilers fed 15% DDGS during the 0–21 days. Recent studies have indicated that increasing the DDGS level in broiler diets to 16% had no negative effect on growth performance on days 0–21 and 22–42 [9, 10]. Although the age of broiler chickens examined in these reports varied, we can be confident that the inclusion of 5% DDGS in the starter period and 10% DDGS in the growing period was safe and did not cause a loss of growth performance. In addition, considering the high levels of anti-nutritional factors (NSP and Phytate) and poor amino acid digestibility in DDGS, supplementation with an exogenous enzyme appears to be a wise choice to ameliorate the negative effect and overcome the limitations of DDGS levels in poultry diets [11].

This study was conducted using corn and soybean meal diets; we expected to reduce anti-nutritional factors and improve nutrient digestibility by supplementing exogenous NSP enzymes, phytase, and protease in C-DDGS and H-DDGS and to investigate the limiting levels of DDGS in broiler diets based on growth performance parameters. The results demonstrated remarkable improvements in BWG and FCR during the grower period (8–28 days) and the overall period (1–28 days) of Cobb broilers when C-DDGS levels (5% in starter and 10% in grower) were supplemented with exogenous multi-enzymes. This result was similar to a report by Abudabos *et al*. [8] indicating that a 6% DDGS diet containing Rovabio® (containing xylanase and β-glucanase; Adisseo, Antony, France) or Tomoko® (containing phytase, protease, and carbohydrase; Adisseo, Tokyo, Japan) exhibited better FI, BWG, and FCR compared with an unsupplemented diet from 0 to 35 days of age. Similar results were also found in a study by Kwak *et al*. [17], in which diets containing 5% or 10% DDGS supplemented with a multienzyme complex (mannanase and xylanase, glucanase) demonstrated higher average daily gain, average daily FI, and feed efficiency during the period of 0–35 days. Furthermore, arabinoxylan are the predominant components of NSP in DDGS, and more studies have focused on the effects of adding xylanase to diets containing DDGS. Liu *et al*. [14] reported that a 10% DDGS diet supplemented with 3600 U/kg xylanase resulted in the greatest increase in BWG. In addition, Swiatkiewicz *et al*. [15] found an increase in BWG in chicks during the 0–21 days when a 12% DDGS diet was supplemented with 1,4-β-xylanase. However, phytase supplementation alone in C-DDGS (10%) supplementation was not beneficial to the growth performance of broilers during 0–21 days of age [30]. This study suggests that increased heat processing during ethanol production could partially destroy or degrade phytate in DDGS. Therefore, using DDGS to replace corn and soybean meal could reduce the concentration of phytate and substrate for phytase, resulting in a reduced effect of phytase in diets [30, 31]. Furthermore, although there are a few reports that C-DDGS diet supplementation with multiple enzymes has no improvement in growth performance [30, 32], the beneficial effect of combining multiple enzymes (xylanase, amylase, protease, and phytase) in corn-soybean meal-based diets has been demonstrated in previous research by Olukosi *et al*. [33] and Amerah *et al*. [34].

Despite using the same enzyme levels, the current study concluded that high dietary DDGS levels (>10%) led to marked depression in growth performance, especially in the second period, exhibiting lower BWG and FI, along with a poorer FCR compared with the conventional diet. Abudabos *et al*. [8] reported that broiler diets supplemented with 12%, 18%, and 24% DDGS negatively impacted FI and BWG and resulted in higher FCR during 0–35 days compared with 6% and non-DDGS diets under the same conditions. The use of enzymes (Rovabio and Tomoko) in diets with high DDGS levels can improve growth performance to a certain extent, but when compared with C-DDGS levels or a DDGS-free diet, all growth performance parameters still exhibit a significant disparity [8]. Campasino *et al*. [7] reported that a 15% DDGS diet decreased body weight on days 7, 14, and 21 and increased FCR during 0–14 days despite NSPase supplementation. Similarly, Khose *et al*. [35] reported negative effects on body weight and FI on 28 days of age in birds fed a 15% DDGS diet (with or without enzyme) compared with those fed a diet containing 5% DDGS. Extreme experiments have shown that diets containing 30% and 60% corn distiller grains resulted in a decrease in FI of 4.1% and 13.7%, respectively while supplementing carbohydrase could increase FI by at least 30 g and also improve weight gain by 17 g of broiler in 15–22 days [36]. We postulated that H-DDGS may impair the growth performance of broilers mainly due to a decrease in FI. In addition, it should be noted that a H-DDGS diet (10% and 20%) supplemented with multienzyme had no significant effect on FI and BWG, and the FCR was numerically lower than that of a C-DDGS enzyme-free diet in the current study. Recent research also concluded that the multicarbohydrase (xylanase, b-glucanase, and ABF) and phytase complex showed the greatest efficacy in increasing BWG and improving FCR when added to the 14% DDGS diet [37]. Swiatkiewicz *et al*. [15] found that the addition of enzyme (xylanase + phytase) to a diet containing H-DDGS (12% in starter and 18% in finisher diet) without any detrimental effect on BWG and FCR during either the 0–21 d or 21–42 d, or the overall feeding period. Based on the results of Swiatkiewicz *et al*. [15] and the current findings, we can conclude that the DDGS level could be increased to 10% in the starter diet and 20% in the grower diet when supplemented with a more comprehensive enzyme, with no adverse effects on the growth performance of broilers.

### Effects of high-level dietary DDGS supplemented with multiple enzymes on the nutrient digestibility of broiler

In the present study, the C-DDGS diet with multienzyme supplementation exhibited higher ileal apparent digestibility of DM, CP, and HC, but no difference was observed in AIDE, fat, and CF content for broiler on 28 days. This finding is consistent with previous research by Olukosi *et al*. [19], who reported that 10% DDGS supplemented with xylanase and protease improved DM and nitrogen use without affecting fat digestibility compared with the control at the ileal level. Similarly, another study using the same level of DDGS reported that supplementing an admixture of xylanase, amylase, and protease increased the digestibility of nitrogen, whereas the addition of phytase alone improved the coefficient of apparent ileal DM digestibility [30]. Recent studies at the same DDGS levels (5% and 10%) as the present study indicated that exogenous mannanase or multienzyme (mannanase and xylanase, glucanase) in a diet containing DDGS resulted in increased DM, CP, and GE digestibility compared with no enzyme-supplemented treatments [17]. Campasino *et al*. [7] reported higher ileal digestible energy and nitrogen digestibility coefficients when NSPase was supplemented with a 5% DDGS diet. However, similar results were not observed in the current study. The main reason may be that the effect of exogenous enzymes on young broilers is more pronounced [33]. In addition, a diet containing 5% DDGS containing commercial protease enhanced CP and amino acid usage in broilers [38].

Furthermore, H-DDGS likely reduce the nutrient digestibility of broilers, probably due to the considerable amount of NSP in DDGS, especially soluble NSP, which can increase the viscosity of intestinal digesta, decrease the interaction times between enzymes and substrates, and interfere with the absorption of nutrients such as fat, protein, and carbohydrates [12, 39]. Our study also observed a significant negative impact on the digestibility of DM and AIDE in 28-day-old broilers when a diet containing 15% and 30% DDGS was fed compared with an enzyme-free diet; however, this effect was not observed in a 10% and 20% DDGS diet. The failure of multienzyme to improve energy digestibility and nitrogen in the 30% DDGS-containing diet is in agreement with previous results by Min *et al*. [40]. However, the digestibility of HC and CF in a 15% and 30% DDGS diet increased by 12.1% and 14.5%, respectively, similar to the results reported by [14]. DDGS usually contains a high content of NSP ranging from 250 to 337 g/kg on a DM basis, and 30% DDGS significantly increased the dietary NSP content by 18% in this study. This increased substrate concentration, especially HC, due to high contents of xylose, arabinose, mannose, and galactose, can be partially degraded by about 9% in pre-cecal by supplementing with NSP enzymes [19]. In addition, it is also well-documented that the protein structure is destroyed and the amino acid content is reduced by the Maillard reaction during heat treatments during the drying process of DDGS, compromising the availability of protein and decreasing the digestibility of most amino acids by 10% units [5, 41]. Kim *et al*. [42] reported that a diet containing 20% DDGS reduced CP digestibility by 1.7%. Our results indicated that a diet with H-DDGS (>10%) reduced the digestibility of CP, but this effect appeared to be mitigated by multienzyme supplementation, as no significant difference was observed between H-DDGS diet and the C-DDGS enzyme-free diet. Overall, this study concluded that supplementing the C-DDGS-level diet with multiple enzymes can improve nutrient digestibility, whereas supplementing a medium-to-high DDGS-level diet with multiple enzymes can alleviate the negative effects of DDGS to a certain extent.

### Effects of high-level dietary DDGS supplemented with multiple enzymes on the intestinal morphology of broiler

The most regarded indicators of intestinal function and health are the intestinal villi height, CD, and villus/crypt [43]. The current study showed that multienzyme supplementation in C-DDGS diet increased VH and the ratio of the entire small intestine but did not affect CD. Karimi *et al*. [44] found that the VH of the duodenum increased with the dietary supplementation of either β-betaglucanase or β-mannanase. Kwak *et al*. [17] reported an increase in jejunal VH in broilers fed a diet containing DDGS and complex enzymes. In addition, supplementation with multiple enzymes in corn and SBM-based diets increases ileal villi height [45] or improve all duodenal morphological parameters [46]. It can be speculated that the improvement in the morphology of different intestinal segments may be the result of a combination of multienzymes, but the effects of different types of enzymes on different intestinal segments still require further validation. However, it is certain that an increase in VH and villus/crypt may indicate an improvement in nutrient digestion and absorption capacity by the intestinal tract, ultimately benefiting growth performance [43]. Furthermore, this study observed a dramatic impact of H-DDGS diet (15% and 30%) on the morphology of various intestinal segments of the small intestine. Similar results were also found in a study by Min *et al*. [47], in which an excess of 15% DDGS resulted in poor intestinal morphology throughout the small intestine. Damasceno *et al*. [9] also observed a linear decrease in jejunum villi height and CD with increasing levels of DDGS from 0% to 16%. Diets containing DDGS can disrupt villus morphology by upregulating the immune response [48], and enzyme supplementation can reconstruct the mucus layer by the reestablishment of goblet cells, thus restoring these harmful effects [49, 50]. This hypothesis was confirmed in a 10% and 20% DDGS diet in this study, in which H-DDGS supplemented with multienzymes showed similar intestinal morphological characteristics to C-DDGS levels with an enzyme-free diet.

### Effects of high-level dietary DDGS supplemented with multiple enzymes on pellet quality and economic benefits

The physical quality of pellets can be evaluated using pellet durability and hardness parameters. To more realistically reflect the conditions of feed used by experimental animals, this study assessed the transportation durability of feed pellets by comparing powder contents from experiment farms and factories. Results showed that diets containing 15%, 20%, and 30% DDGS dramatically reduced feed hardness and transportation durability, whereas diets containing 5% and 10% DDGS showed no difference. Similar results were observed by Shim *et al*. [3], who reported that diets containing DDGS >15% had a harmful effect on pellet durability. Kim *et al*. [42] also found a significant decrease in pellet durability and hardness in diets containing 20% DDGS. Diets containing H-DDGS usually require large amounts of oil supplementation to balance dietary energy levels, resulting in a higher fat concentration in the diet and worse pellet physical quality [51]. The hydrophobic nature of fats interferes with water in the form of water vapor, degrading the natural bonds in the feed pellets and affecting their binding. Dietary added fat reduces pelletizing pressure on the mold due to its lubricating properties, which may further reduce pellet quality [52]. In addition, H-DDGS in the diet usually have a lower starch content due to the lower starch content in DDGS, which could result in less starch gelatinization and reduced particle binding [53]. Although NSP enzymes are thought to degrade plant cell walls by breaking glycosidic bonds, the release of starch particles inside the cells may contribute to adhesion between particles in pellets, thereby increasing feed pellet quality [54]. However, this was not verified in this experiment. Furthermore, studies have shown that pelleted diets are more beneficial for BWG and FI than mash diets, and feeding diets with relatively smaller particle sizes can reduce the FI and gizzard function of broiler chickens [55]. In addition to reduced nutrient digestibility caused by impaired intestinal morphology, decreased FI caused by high dietary powder content may be another cause of slow weight gain in high-DDGS diet treatment.

The high sulfur concentration in the DDGS cannot be ignored. This occurs because sulfuric acid is added during ethanol production to maintain the pH and activity of yeast, and ultimately, sulfate ions are retained in DDGS. The sulfur content of DDGS from different sources varies greatly, usually between 6000 and 10,000 ppm [56]. In this study, the sulfur content was 9042 ppm, and the highest sulfur content in the 30% DDGS diet was 4470 ppm. The NRC [57] concluded that sulfur toxicity is 14,000 ppm in chicks (reduced growth) and 8100 ppm in laying hens (reduced egg production). Thus, the sulfur content of the diet used in this study is considered safe. Although a 30% diet significantly increased dietary sulfur levels, they remained within the safe range.

Finally, the economics of DDGS are the primary reason for using it as a substitute for corn and soybean meal in broiler diets [2]. In this study, the cost of the two high-level DDGS with multienzyme diets decreased by 0.0077 and 0.0041 USD/kg, respectively, compared with the C-DDGS diet, indicating that the use of DDGS greatly helped reduce the cost of the diet. Despite the increased enzyme cost, the final unit meat production cost of each treatment group using multiple enzymes was reduced to varying degrees, with the 10% and 20% DDGS diet exhibiting the highest economic benefits.

## Conclusion

The results of the current study demonstrated that the C-DDGS diet supplemented with multienzyme improved growth performance by enhancing nutrient digestibility and increasing the intestinal morphology of broiler chickens from 0 to 28 days of age. Diets containing H-DDGS (15% in starter and 30% in grower) reduce FI and nutrient digestibility by affecting intestinal morphology and feed pellet quality, ultimately hindering weight gain. In addition, when supplemented with multienzyme, dietary DDGS can be safely included at levels of 10% in 0–7 days and 20% in 8–28 days without impairing growth performance compared with C-DDGS enzyme-free diet, and this approach yields the highest economic benefit.

## Author’s Contributions

DJ and II: Conceived and designed the study and drafted the manuscript. DJ and ET: Performed the experiments. EAR and RR: Performed statistical analyses. TW and AS: Samples analysis and manuscript editing. All authors have read, reviewed, and approved the final manuscript.
